# Efficacy of Visual Surveys for White-Nose Syndrome at Bat Hibernacula

**DOI:** 10.1371/journal.pone.0133390

**Published:** 2015-07-21

**Authors:** Amanda F. Janicki, Winifred F. Frick, A. Marm Kilpatrick, Katy L. Parise, Jeffrey T. Foster, Gary F. McCracken

**Affiliations:** 1 Department of Ecology and Evolutionary Biology, University of Tennessee, Knoxville, Tennessee, United States of America; 2 Department of Ecology and Evolutionary Biology, University of California Santa Cruz, Santa Cruz, California, United States of America; 3 Center for Microbial Genetics and Genomics, Northern Arizona University, Flagstaff, Arizona, United States of America; Università degli Studi di Napoli Federico II, ITALY

## Abstract

White-Nose Syndrome (WNS) is an epizootic disease in hibernating bats caused by the fungus *Pseudogymnoascus destructans*. Surveillance for *P*. *destructans* at bat hibernacula consists primarily of visual surveys of bats, collection of potentially infected bats, and submission of these bats for laboratory testing. Cryptic infections (bats that are infected but display no visual signs of fungus) could lead to the mischaracterization of the infection status of a site and the inadvertent spread of *P*. *destructans*. We determined the efficacy of visual detection of *P*. *destructans* by examining visual signs and molecular detection of *P*. *destructans* on 928 bats of six species at 27 sites during surveys conducted from January through March in 2012–2014 in the southeastern USA on the leading edge of the disease invasion. Cryptic infections were widespread with 77% of bats that tested positive by qPCR showing no visible signs of infection. The probability of exhibiting visual signs of infection increased with sampling date and pathogen load, the latter of which was substantially higher in three species (*Myotis lucifugus*, *M*. *septentrionalis*, and *Perimyotis subflavus*). In addition, *M*. *lucifugus* was more likely to show visual signs of infection than other species given the same pathogen load. Nearly all infections were cryptic in three species (*Eptesicus fuscus*, *M*. *grisescens*, and *M*. *sodalis*), which had much lower fungal loads. The presence of *M*. *lucifugus* or *M*. *septentrionalis* at a site increased the probability that *P*. *destructans* was visually detected on bats. Our results suggest that cryptic infections of *P*. *destructans* are common in all bat species, and visible infections rarely occur in some species. However, due to very high infection prevalence and loads in some species, we estimate that visual surveys examining at least 17 individuals of *M*. *lucifugus* and *M*. *septentrionalis*, or 29 individuals of *P*. *subflavus* are still effective to determine whether a site has bats infected with *P*. *destructans*. In addition, because the probability of visually detecting the fungus was higher later in winter, surveys should be done as close to the end of the hibernation period as possible.

## Introduction

Disease surveillance in wildlife is often limited by diagnostic techniques that are cost-effective, rapid, and feasible for use on wild animals [1, 2]. For diseases where hosts display visible symptoms, visual surveys are often cost-effective and can be appealing for surveillance because they typically impose minimal disturbance on host populations [3, 4]. However, if hosts have cryptic infections that are not observable, then visual surveys will have limited utility for reliably identifying habitats harboring infected individuals (a primary goal of disease surveillance) and will underestimate infection prevalence. Estimating the efficacy of visual surveys for a particular disease is necessary to determine whether this low-cost and minimally disruptive survey method is an appropriate surveillance approach.

White-Nose Syndrome (WNS) is a rapidly spreading epizootic disease that has caused widespread declines in six species of hibernating bats in North America, raising substantial concern about the risk of extirpation and extinction of species [5–8]. WNS is caused by the fungal pathogen, *Pseudogymnoascus destructans* [9–11], which infects and kills bats during hibernation [12] by disrupting physiology [13–15] and natural torpor arousal patterns [10, 16]. The disease was named WNS because the faces and wings of some initially documented bats were visibly covered in white, powdery fungal growth [17]. The disease was first detected in a cave near Albany, New York in 2006, and by the spring of 2015 WNS had been confirmed in seven species of bats in 26 U.S. states and five Canadian provinces [18]. Although the exact origin of *P*. *destructans* remains unclear, recent genetic data suggest the fungus was introduced to North America from the Western Palearctic [19, 20].

Visual surveillance for WNS is conducted in hundreds of caves and mines each year and is the primary surveillance strategy recommended by the U.S. Fish and Wildlife Service WNS National Response Plan and the Canadian Wildlife Heath Cooperative WNS National Plan [21, 22]. Surveillance for WNS consists primarily of searching for bats with visible fungal infections of *P*. *destructans* (e.g. visible fungus on skin tissues), and submitting bats with suspected infection for laboratory testing by histopathology [23]. Histopathology is used to confirm the presence of epidermal cupping erosions and lesions on the wing membrane diagnostic of WNS disease [23]. Reporting of hibernacula with WNS is used to track disease spread as well as inform management decision-making, such as restricting human access to sites or requiring decontamination protocols to reduce potential spread of the fungus by humans [24].

Bats become infected with *P*. *destructans* before the fungus on skin tissues becomes visible to the human eye. These cryptic infections could easily be missed during visual surveys, causing sites to be falsely classified as ‘uninfected’ when in fact the pathogen is present and bats are infected. Falsely reporting a site as not having bats infected with *P*. *destructans* could lead to underestimates of the impact of disease on bat populations, and unrestricted human access without decontamination could lead to inadvertent spread of *P*. *destructans*. False visual detections of *P*. *destructans* caused by other fungi such as *Trichophyton redellii* [25] could also occur and could lead to unnecessary killing of bats for submission for histopathology. The recent development of a qPCR assay [26] to detect *P*. *destructans* DNA from epidermal swab samples from bats provides an opportunity to determine the accuracy and efficacy of visual surveys for detecting the presence of the pathogen at hibernacula and the prevalence of infection on different bat species. Although a range of different factors can affect DNA quantity extracted from swabs (e.g. extraction efficiency), this qPCR assay has been shown to be both highly specific to *P*. *destructans* and highly sensitive, making it an accurate and useful method to determine if bats are infected and for estimating prevalence [27, 28].

Our main objective was to determine the accuracy of visually detecting infections of *P*. *destructans* at bat hibernacula. Here, we define infection as the presence of *P*. *destructans* DNA detected by qPCR from swab samples collected from bats. We estimated the probability of failing to visually detect infections on bats that tested positive for *P*. *destructans* by qPCR (i.e. the probability of an infection being cryptic). We hypothesized that cryptic infections would be less likely in bats with higher pathogen loads, and as a result, cryptic infections would be more likely in species with lower pathogen loads [12]. We also compared whether the presence or absence of particular bat species at a hibernaculum increased the probability of visually detecting *P*. *destructans* on bats.

## Materials and Methods

### Sample collection

**Fig 1:**
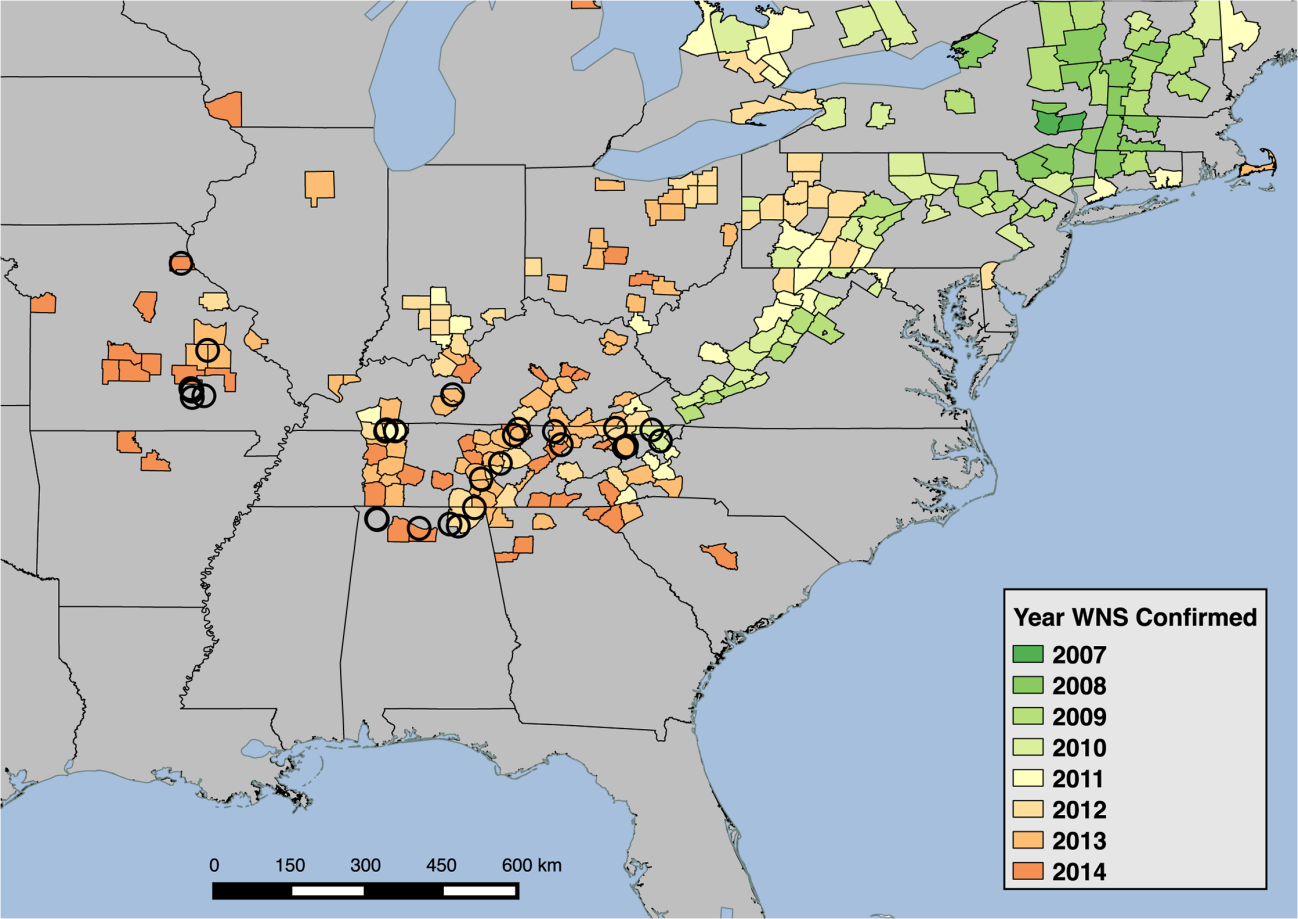
Map of sample collection. A map of 27 hibernacula in four states where hibernating bats were sampled from January-March in 2012–2014. Shading designates the year that WNS and molecular evidence of *P*. *destructans* were confirmed in a U.S. county [18].

We examined the presence of *P*. *destructans* in six species in 27 hibernacula in four states in the southeastern United States (Fig 1) during winter hibernation from January through March in 2012–2014. We swabbed 928 bats of six species over three years with an average of 22 bats (range: 5–50) of one to six species present in each hibernaculum. Bats were swabbed five times on their muzzle and forearm with polyester-tipped swabs dipped in sterile water. Prior to swabbing, we noted whether fungus was visible on the bat’s skin tissues (muzzle, ears, forearms, and uropatagium) while the bat was in hand. All bats were released after sampling at the site where they had been roosting. Swabs were stored in RNAlater to preserve DNA and kept refrigerated or frozen until testing.

All bat handling procedures followed guidelines approved by the American Society of Mammalogists and the University of Tennessee Institutional Animal Care and Use Committee. Decontamination procedures issued by U.S. Fish and Wildlife Service were followed for all caving gear [24]. Permits for this research were obtained from Missouri Department of Conservation (15184, 15471, and 15871), Tennessee Wildlife Resources Agency (3716), and U.S. Fish and Wildlife Service (TE71613A-0). Other bat samples were collected in collaboration with state agency personnel with permits from Alabama Wildlife and Freshwater Fisheries and Kentucky Department of Fish and Wildlife Resources.

### Sample testing

Swab samples and standards were extracted with DNeasy Blood and Tissue extraction kits (Qiagen, Valencia, CA) with modifications for fungal extractions that included the addition of lyticase during the lysis step [28]. Each extraction plate had 16 negative control wells (100% *P*. *destructans* negative) distributed throughout the plate. DNA samples were analyzed by real-time PCR using methods developed by Muller et al. [26], using a cut-off of 40 cycles for a positive detection. Cycle threshold values (Ct value) were used to calculate fungal loads, in nanograms, using the equation load = 10^((22.04942-Ct value)/3.34789)^, which was derived from serial dilutions of a quantified standard of isolate *P*. *destructans* 20631–21. Seventy-five percent of samples were run in duplicate and a sample was considered *P*. *destructans* positive if either or both runs were positive. Fungal loads were averaged across both runs after conversion from Ct values.

### Statistical analysis

#### Visual detection of *P*. *destructans* on bats

We used generalized linear models with a binomial distribution to determine if the probability of visually detecting *P*. *destructans* on a bat was associated with fungal load, when sampling occurred, and if detection probability differed among species. We used a bias-reduction method (package brglm in R v. 3.1.2) to deal with the complete separation present in the data (in some species no visual detections of the fungus were made). We used the number of days since January 1^st^ to account for differences in timing of sampling as visibility of infection may increase later in the season [27, 29, 30]. We fit twelve *a priori* models with combinations of main, additive, and interactive effects representing our hypotheses and used Akaike information criterion (AIC) model selection criteria to determine the best-fitting model. We estimated the probability of falsely detecting visual infection using bats that tested negative by qPCR but were noted with visible white fungus in the field. We compared whether false detection differed among species using a likelihood ratio test to compare a null model to one with species included.

#### Visual surveys for site-level detection of *P*. *destructans* on bats

We used generalized linear models with binomial distributions in which each site visit was a data point to determine whether timing of survey, sampling effort, and species of bats examined influenced visual detection of *P*. *destructans* on bats at a site. For *Myotis lucifugus*, *Myotis septentrionalis*, and *Perimyotis subflavus*, we also determined whether the prevalence of infection of bats with visual infections influenced the likelihood of visually detecting the fungus during a site visit. All statistical analyses were conducted in Program R v. 3.1.2.

## Results

### Pathogen loads and visual detection of *P*. *destructans* on bats

**Fig 2:**
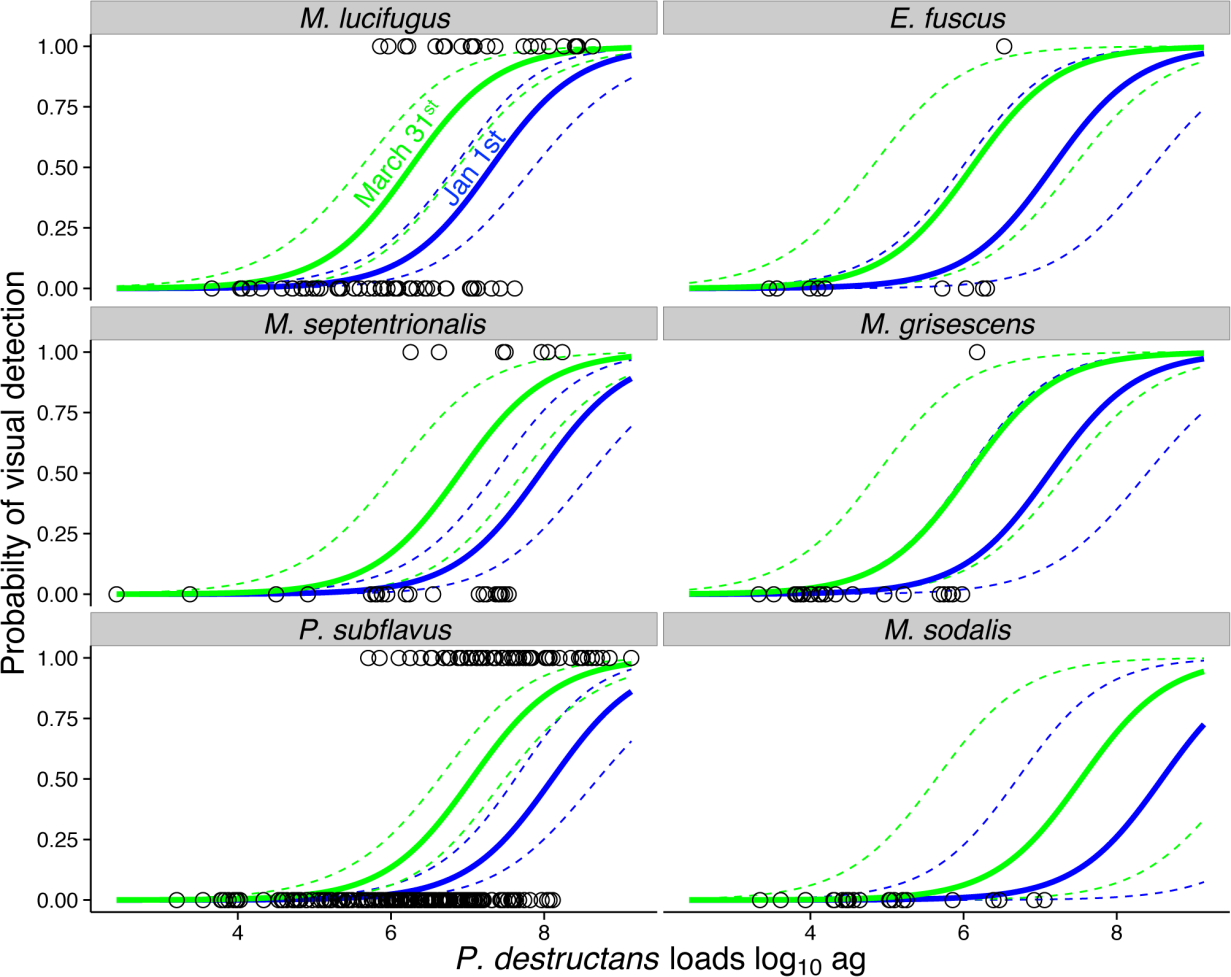
Visual detectability of *Pseudogymnoascus destructans* on bats compared to fungal loads. Solid lines show predicted relationships from the best-fit model (Table 2) and dashed lines show the 95% confidence bands for early (January 1^st^; blue lines) and late (March 31^st^; green lines) sampling dates. Individual circles are bats that tested positive for *P*. *destructans* by qPCR and did (y-axis value of 1) or did not (0) have visible evidence of *P*. *destructans*.

Seventy-seven percent (306/397) of bats that tested positive for *P*. *destructans* by qPCR had no visible signs of *P*. *destructans*, demonstrating that the probability of false negatives (i.e. failing to visually detect *P*. *destructans* on bats that had the pathogen) is high (Table 1). The probability of observing visible white fungus on a bat that was qPCR negative was low (14/531 or 2.6%) and did not differ among species (likelihood ratio test: χ = 5.10, df = 5; *P* = 0.40). The best-fitting model of the probability of visual detection included fungal load, sampling date, and an additive species effect (AIC weight = 0.55; Fitted equation for *M*. *lucifugus* = Pr(Detection) ~ -12.9 (±1.5) + 1.77 (±0.2) * log_10_(load) + 0.02 (±0.01) * (days since January 1); For *M*. *septentrionalis* and *P*. *subflavus* the intercept equaled -14.01 (±1.6); For the three other species (*Eptesicus fuscus*, *Myotis grisescens*, and *Myotis sodalis*) the intercept equaled -13.47 (±1.6)), suggesting that the probability of visually detecting *P*. *destructans* on a bat increased with pathogen load measured by qPCR, but the slope did not differ among species (Table 2, Fig 2). The probability of visually detecting *P*. *destructans* increased with the number of days since January 1^st^ and there was only weak support that this effect differed among species (Table 2).

**Table 1:** Fraction of bats with visible fungus on bats tested for *Pseudogymnoascus destructans* by qPCR.

Species	Fraction of bats with visible fungus	
	qPCR +	qPCR -
*Eptesicus fuscus*	0.10 (1/10)	0.0 (0/30)
*Myotis grisescens*	0.04 (1/26)	0.02 (5/201)
*Myotis lucifugus*	0.35 (24/69)	0.06 (3/50)
*Myotis septentrionalis*	0.24 (7/29)	0.0 (0/22)
*Myotis sodalis*	0.0 (0/21)	0.05 (4/76)
*Perimyotis subflavus*	0.24 (58/242)	0.01 (2/152)

Sample sizes are shown in parentheses.

**Table 2:** Model selection results for visual detectability of *Pseudogymnoascus destructans* on bats.

Model	ΔAIC	AIC weights
**species + load + date**	0.0	0.55
species * date + load	2.0	0.20
species + date * load	2.1	0.19
species + load	5.2	0.04
load	7.1	0.02
species * load + date	9.7	0.00
species * load	18.3	0.00
species * load * date	29.0	0.00
species + date	118.8	0.00
species	128.6	0.00
date	132.4	0.00
pd.visible.bat ~ null	142.0	0.00

Models are ranked by ΔAIC and the best-fitting model is shown in bold.

Visible infections occurred most frequently in three species (*M*. *lucifugus*, *M*. *septentrionalis*, and *P*. *subflavus*) that had the highest fungal loads and *M*. *lucifugus* had a significantly lower detectability threshold (e.g. higher intercept) compared to *M*. *septentrionalis* and *P*. *subflavus*, which were not significantly different from each other (Fig 2). Loads on the other three species (*E*. *fuscus*, *M*. *grisescens*, and *M*. *sodalis*) were usually too low to result in visible infection (Fig 2).

### Efficacy of visual surveys at hibernacula

**Fig 3:**
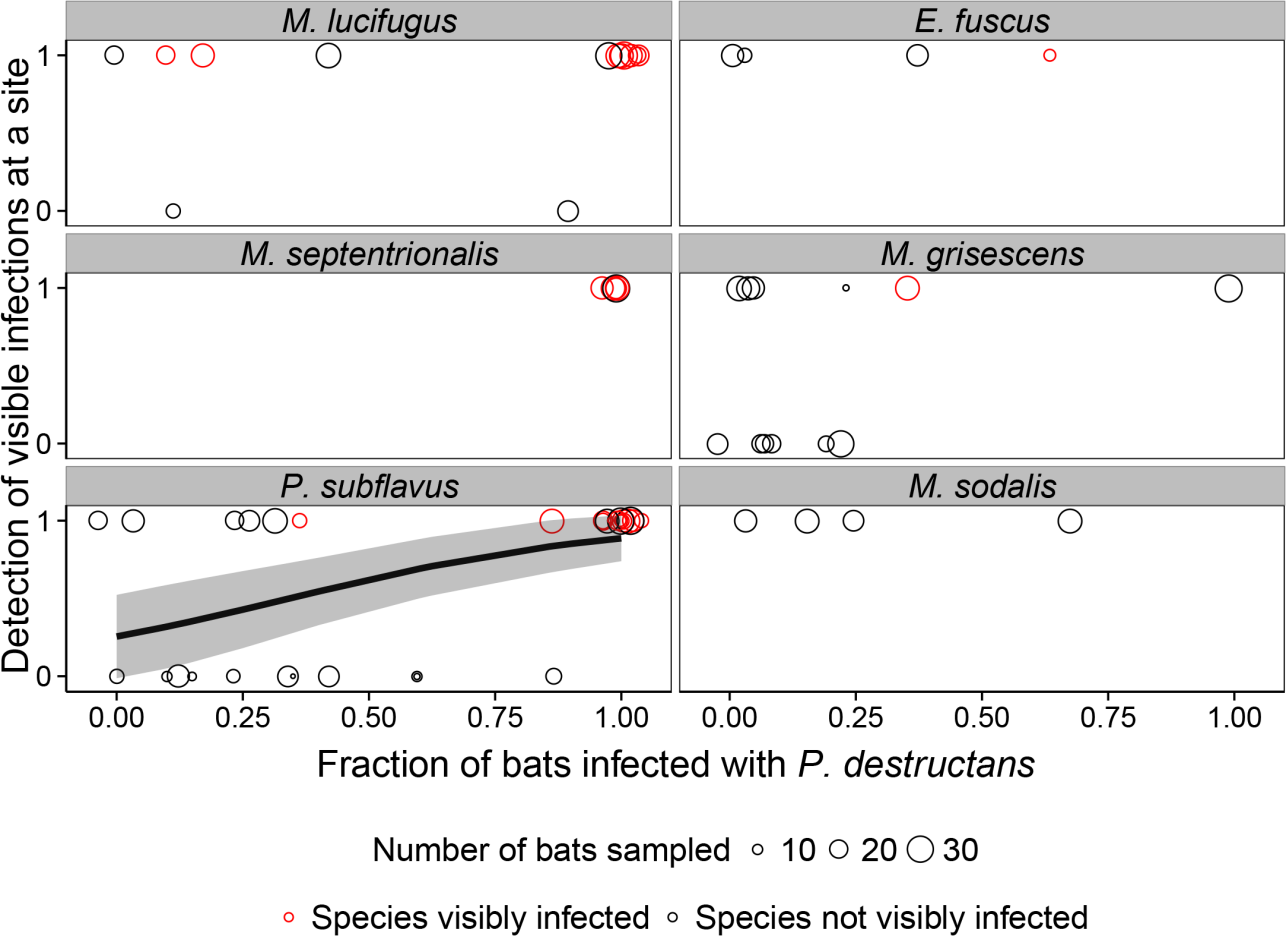
Detection of visible *Pseudogymnoascus destructans* on bats at hibernacula and the fraction of bats with *P*. *destructans* at that site as determined by qPCR. Circles represent sites where a species was sampled, with red circles indicating sites where at least one individual of that species had visible fungus and black circles indicating sites where no individuals of that species were observed with visible fungus. The size of the circles is scaled to the number of bats sampled at a site. The x-axis shows the proportion of bats that were positive for *P*. *destructans* by qPCR and the y-axis shows whether at least one individual bat at that site of any species was negative (0) or positive (1) for visible fungal infections. Prevalence of infection was a significant predictor for detection of visible infections at a site for a single species, *P*. *subflavus*. Solid black line and gray shading for *P*. *subflavus* represent the best-fit line and 95% confidence band for the relationship between prevalence of infection and detection of visible infections at hibernacula.

Forty percent (17/43) of sites where at least one bat tested positive for *P*. *destructans* by qPCR had no bats with visual signs of *P*. *destructans* and would have been classified as ‘uninfected’ based solely on visual surveys. The likelihood of detecting the presence of *P*. *destructans* at a site with visual surveys increased with the number of bats examined for the three species that frequently exhibit visual infections (*M*. *lucifugus*, *M*. *septentrionalis*, and *P*. *subflavus*) (Pr(Detection) ~ -0.90 + 0.12 (± 0.051) * #mylu.myse.pesu.sampled; N = 43; *P* = 0.02), and there was very weak support for the influence by when a visit occurred between January and March or examination of other species (Table 3). The probability of visual detection of *P*. *destructans* at a site increased with prevalence of infection for *P*. *subflavus* (Pr(Detection) ~ -1.1 + 3.13 (± 0.051) * Prevalence; N = 32; *P* < 0.01), but not for *M*. *lucifugus* (Pr(Detection) ~ -1.1 + 1.0 (± 1.8) * Prevalence; N = 13; *P* = 0.56) or *M*. *septentrionalis*, the latter of which had a prevalence of 100% at all sites (Fig 3). Visual surveys that include either 17 *M*. *lucifugus* or 17 *M*. *septentrionalis* have a 99% likelihood of detecting *P*. *destructans* if it is present at the site. For *P*. *subflavus*, examining at least 29 bats is required to have a 99% chance of detecting *P*. *destructans* if it is present.

**Table 3:** Model selection results for visual detectability of *Pseudogymnoascus destructans* at hibernacula. #mylu.myse.pesu.sampled refers to the sum of the number of bats of three species sampled (*M*. *lucifugus* – mylu, *M*. *septentrionalis* – myse, *P*. *subflavus* – pesu).

Model	ΔAIC	AIC weights
**#mylu.myse.pesu.sampled**	0.0	0.56
date + #mylu.myse.pesu.sampled	1.3	0.29
null	4.9	0.05
all.bats.sampled	5.0	0.05
all.bats.sampled + date	5.6	0.03
date	6.6	0.02

Models are ranked by ΔAIC and the best-fitting model is shown in bold.

## Discussion

Our results suggest that cryptic infections are widespread and that solely using visible signs of *P*. *destructans* greatly underestimates infection prevalence in bats even during mid to late winter (January-March) when the majority of surveillance surveys for WNS are conducted. Cryptic infections were so common in some species (*E*. *fuscus*, *M*. *grisescens*, and *M*. *sodalis*) that visual surveys were only useful for detecting *P*. *destructans* at a site if other species (*M*. *lucifugus*, *M*. *septentrionalis*, and/or *P*. *subflavus*) also were present and examined. The higher percentages of the latter three species that displayed visible *P*. *destructans*, combined with the high infection prevalence in these species, resulted in a very high likelihood that *P*. *destructans* was detected at a site whenever these bat species were present.

Our results also show that the probability of visual detection increases with fungal load of *P*. *destructans*, and differences in fungal loads among species explain most of the differences in the probability of observing visible *P*. *destructans* on bats. This is likely because higher loads indicate a larger number of conidia and hyphae on the bats and a greater likelihood of the fungus being visible. This is consistent with the finding that the probability of visual detection of *P*. *destructans* on bats was higher later in the hibernation season when the fungus has had sufficient time to grow on the bats and is at maximal loads [12], suggesting that visual surveys should be scheduled late in hibernation to be maximally effective. Our findings are similar to patterns of visual prevalence in Europe where visible infections also peaked in late hibernation [29]. Hibernation season length may influence visual detection given that most bats become infected at the start of hibernation and fungal loads increase once bats are torpid [12]. Thus, infections may become visible sooner in northern latitudes where bats likely enter hibernation earlier [31].

Even with the same fungal load, some species were more likely to exhibit visible *P*. *destructans* (Fig 2). Visible *P*. *destructans* was detected at significantly lower loads on *M*. *lucifugus* than other species (Fig 2), perhaps because their darker skin provides more visual contrast with the white fungus. *Myotis lucifugus* and *M*. *septentrionalis*, when present, are the best ‘sentinels’ or indicators of the presence of *P*. *destructans* when surveying for visible signs, and surveying *P*. *subflavus* can also be useful. In contrast, fungal loads in *E*. *fuscus*, *M*. *grisescens*, and *M*. *sodalis* are simply too low to consistently result in visible *P*. *destructans*. Differences in fungal loads and infection intensity among species suggests interesting differences in either transmission, hibernating behaviors, and/or disease susceptibility among hibernating species exposed to *P*. *destructans* [5, 32, 33].

Currently, visual surveys are routinely used to determine whether *P*. *destructans* has invaded new hibernacula [21, 22]. Our results show that the efficacy of these visual surveys depends on which species are present at a site and how many bats are examined for visible fungus. For example, the presence of *M*. *lucifugus* or *M*. *septentrionalis* increases the probability that *P*. *destructans* can be detected visually at a site and that these can be used as ‘sentinel’ species for the presence of *P*. *destructans* (Fig 3). Our results suggest that with a moderate survey effort of examining either 20 (if surveying *M*. *lucifugus* or *M*. *septentrionalis*) or 30 (if only *P*. *subflavus* are examined) individuals at a site, then visual surveys can indeed be effective at determining whether bats are infected with *P*. *destructans* at a hibernaculum. At sites with species that rarely or never have visible signs of *P*. *destructans*, such as *E*. *fuscus*, *M*. *grisescens*, or *M*. *sodalis*, visual surveys are ineffective. To ensure visual surveillance is effective at determining whether *P*. *destructans* has invaded new sites [21, 22], future surveillance guidelines should incorporate these specific recommendations on species and sample sizes required for effective surveillance efforts.

The widespread occurrence of cryptic infections in all species has direct relevance to management and surveillance of this disease [34, 35]. Visual surveys can be an effective and relatively low-cost part of surveillance activities, especially in areas where routine winter colony counts are already conducted [36], only as long as sites contain sufficient numbers of bats (>20) of species that exhibit visual infections (e.g. *M*. *lucifugus*, *M*. *septentrionalis*, and/or *P*. *subflavus*). Further, visual surveys of individual bats are most effective late in the hibernation season. However, for detection of *P*. *destructans* on species with predominately cryptic infections and to accurately measure prevalence, swab sampling and testing samples with molecular methods are needed [12, 26, 27]. Ultraviolet (UV) illumination has recently been proposed for WNS surveillance based on comparisons with histological examination of bats submitted for testing based on visual signs of WNS and bats collected in areas where the fungus has been present for several years [37]. We did not examine bats under UV illumination and a comparison of this method with molecular testing of swab samples would be useful to determine whether UV illumination is effective for detecting cryptic infections on the leading edge of fungal invasion. Currently, the U.S. Fish and Wildlife Service WNS National Response Plan and the Canadian Wildlife Health Cooperative WNS National Plan surveillance protocols rely entirely on visual surveillance [21, 22], but our findings suggest that combining swab sampling and visual surveys would improve national surveillance of this disease.

There are currently no active management strategies for control or mitigation of WNS other than cave closures [21, 34]. However, activities such as culling have been considered as a means to prevent the spread of the disease to new regions [38]. The occurrence of cryptic infections demonstrates that culling visibly infected bats will be ineffective at halting the spread of *P*. *destructans*, supporting early modeling efforts [38]. Further, recent evidence suggests that culling infected individuals, even using a highly sensitive method (e.g. qPCR on swab samples), will be ineffective because *P*. *destructans* is often widespread in the environment a year after the fungus reaches a site, and can persist at sites and in the absence of bats for long periods [27, 38–41]. Our findings that cryptic infections commonly occur at bat hibernacula suggest that although the spread of *P*. *destructans* across North America is consistent with spread by bats [42–44], restricting recreational access and requiring field hygiene protocols to decontaminate gear will reduce potential human-mediated spread.
